# Exploration of mental health stigma in veterinary teams in the United States before and after evidence‐based psychoeducation for burden transfer

**DOI:** 10.1002/vro2.84

**Published:** 2024-09-25

**Authors:** Kylie R. Gannon, Elizabeth Cousins, John Martin, Alanna S. G. Updegraff, Christopher Was, Mark D. Carlson, Mary Beth Spitznagel

**Affiliations:** ^1^ Department of Psychological Sciences Kent State University Kent Ohio USA; ^2^ Stow Kent Animal Hospital Kent Ohio USA

## Abstract

**Background:**

High rates of psychological distress are present in veterinary healthcare professionals and elevated mental health stigma in the field may underlie psychological treatment reluctance. A psychoeducational programme designed to reduce distress associated with difficult veterinary client behaviours (i.e., ‘burden transfer’) showed reduced stress and burnout in veterinary teams. We hypothesised that exposure to this psychoeducation could also yield reduced mental health stigma.

**Methods:**

Data from 143 veterinary healthcare professionals who were randomised to intervention (*n* = 72) or control (*n* = 71) groups were examined. Intervention participants completed three weekly 1‐h psychoeducation sessions. Mental health stigma was assessed at pre‐test, post‐test and 1‐month follow‐up.

**Results:**

Psychoeducation decreased mental health stigma in the intervention group relative to controls (*β* = ‒0.28, *p* = 0.009). The participants in this study self‐selected to enroll; thus, the results may reflect attitudes of individuals who were more psychologically open at baseline.

**Conclusion:**

Exposure to evidence‐based psychoeducation, to reduce burden transfer, reduced mental health stigma in veterinary teams.

## INTRODUCTION

A large body of published studies has demonstrated that veterinary healthcare professionals, across various positions in the field, experience elevated psychological distress.^1^, ^2^, ^3^, ^4^, ^5^ High rates of mental health stigma (MHS) are also present in veterinary teams,^6^, ^7^ including reduced belief in the effectiveness of treatment.^3^ The presence of MHS is well known to be linked with avoidance or delay of seeking professional help for mental health problems^8^ and is posited as a key reason for low rates of mental health treatment observed in veterinary teams.^1^, ^2^

A variety of approaches to reduce MHS have been attempted in healthcare providers. Interventions typically utilise direct methods such as education about MHS, social contact with individuals who are diagnosed with mental illness and anti‐stigma campaigns.^9^ Less well studied is the degree to which indirect methods, such as simply exposing individuals to effective and evidence‐based psychological or psychoeducational interventions, might enhance beliefs in treatment effectiveness. If one experiences improvement in mental health or wellbeing as the result of an evidence‐based intervention, intuitively, it might influence their belief in treatment effectiveness for mental health outcomes in general.

It is thus possible that effective workplace psychoeducational programme aimed at reducing distress for veterinary teams would also reduce MHS. A systematic review showed that the evidence‐based acceptance and commitment therapy/training framework (ACT) successfully reduced psychological distress through workplace educational interventions.^10^ Our group created an ACT‐based psychoeducational programme tailored specifically for veterinary medicine, with the goal of helping veterinary teams reduce burden transfer, or reactivity to, the common stressor of difficult client interactions. A randomised controlled trial showed that this programme reduced stress and burnout for participants relative to baseline and a control group.^11^

Although the primary goal of that psychoeducational programme was to reduce burden transfer, stress and burnout, we posited for the current ancillary work that exposing veterinary healthcare teams to this evidence‐based psychological framework might also facilitate positive perceptions of mental health treatments in a more general manner. For the current study, we hypothesised that veterinary healthcare team members who completed the ACT‐based intervention would demonstrate lower MHS relative to their baseline and to a waitlist control group.

## METHODS

The local Institutional Review Board approved the study as a randomised, parallel arms trial. The original trial was conducted and reported^11^ in accordance with CONSORT guidelines.^12^ This ancillary exploration describes a separate hypothesis and outcome from the original trial that was not previously reported.

### Participants

Staff from 17 veterinary clinics, including small animal general hospitals, emergency and referral clinics, and a large academic centre in the Midwestern region of the United States, were invited to participate. The participants were required to be over 18 years old and able to speak and comprehend English. The exclusion criteria included working in a position involving no client interaction.

### Measures

Mental health stigma was measured using the attitudes towards mental illness scale.^13^, ^14^, ^15^, ^16^ This tool is comprised of responses to two statements. First, ‘Treatment can help people with mental illness lead normal lives’; second, ‘People are generally caring and sympathetic to people with mental illness’. Responses are given on a five‐point Likert‐type scale: agree strongly, agree slightly, neither agree nor disagree, disagree slightly or disagree strongly. This brief scale has been implemented in multiple previous studies in various populations and was employed in the present study due to its use in landmark studies examining presence of MHS in veterinary healthcare teams.^2^, ^3^ While some prior uses of this tool adapted it, replacing ‘people’ with ‘veterinarians’, the current study maintained fidelity to the original measure due to our desire to examine this issue across multiple employment positions across veterinary medicine. In the current study, responses on this measure were examined both as percent agreement/neutral/disagreement on each item (for descriptive purposes) as well as summed across items with a higher total reflecting greater MHS (for analytic purposes).

Additional data participants self‐reported demographic information, including age, race, gender, nature of employment and length of time in the profession.

### Psychoeducational programme

The psychoeducational programme utilised in the current study has been previously described^11^, ^17^ and will be briefly summarised here. The programme was tailored specifically for veterinary medicine, focusing on identifying and reducing reactivity to difficult client interactions (that is, ‘burden transfer’) through use of an ACT framework. Techniques to help veterinary teams be more resilient in this context were modelled in the group setting. The programme was delivered through three sessions, 1 h each, spaced 1 week apart, with homework to practice skills assigned between sessions. The programme was interactive and delivered live, online, via video conferencing.

### Procedure

The study was conducted between January and November 2021. Study participants were recruited from attendees of the psychoeducational programme, which was offered in veterinary clinics as a continuing education opportunity. Programme attendees who also agreed to participate in research were randomised to intervention or control. All participants completed measures at a ‘pre‐test’ baseline, ‘post‐test’ within 2 weeks of completing the programme and 1‐month ‘follow‐up’. All the data were collected online via Qualtrics (www.qualtrics.com). Pre‐test measures began with an informed consent. The survey was otherwise the same for all three time points.^11^ Control participants were offered the same programme after completion of the study. Participants were reimbursed via gift card for each survey completed.

### Statistical analyses

Statistical analyses were conducted using Lavaan, a commercially available R software package.^18^ All variables used in the statistical analyses were evaluated for normality using histograms and skewness/kurtosis to ensure that the parametric assumptions were met. Demographic information was characterised using descriptive statistics (percent for categorical data, and mean, standard deviation and minimum–maximum for numeric data). Intervention and control groups were compared utilizing *t*‐tests and chi‐square tests to detect any potential demographic differences across conditions. MHS descriptives for those with complete data at each timepoint were determined using mean and standard error for numeric data (overall MHS score) and percent frequency (categorical response: agree/neutral/disagree). Latent growth curve analysis was used to examine the effect of the programme on MHS in the full sample; multiple imputation of missing data was accomplished with the R multivariate imputation via chained equation package.

## RESULTS

### Participants

The full sample included 143 client‐facing veterinary healthcare team members (intervention, *n* = 72; control, *n* = 71). Across both groups, the sample was comprised primarily of white (96%) females (90%) with an average age of 37 years. The average number of years of employment was 12. Position in the field included veterinarians (18%), technicians (38%), assistants (12%), customer service representatives (13%) and management (12%), with the remaining participants (6%) working in ‘other’ client‐facing roles. No significant demographic differences were detected between intervention and control participants. See Table 1 for demographics.

**TABLE 1:** Participant demographics (*n* = 143).

	Intervention group (*n* = 72)	Control group (*n* = 71)
Age (years)		
Mean standard deviation	37.2 (10.4)	37.5 (11.2)
Range	19‒62	20‒63
Race ethnicity		
White	71 (98.6)	66 (93.0)
Hispanic or Latino	1 (1.4)	2 (2.8)
Black or African American	0 (0.0)	1 (1.4)
Other	0 (0.0)	2 (2.8)
Gender		
Female	65 (90.3)	64 (90.1)
Male	6 (8.3)	8 (8.5)
Prefer not to say	1 (1.4)	1 (1.4)
Experience (years)		
Mean standard deviation	12.5 (9.4)	10.6 (8.6)
Range	1‒40	1‒33
Position		
Veterinarian	19 (26.4)	7 (9.9)
Technician	24 (33.3)	30 (42.2)
Assistant	8 (11.1)	9 (12.7)
Customer service	8 (11.1)	11 (15.5)
Management	7 (9.7)	10 (14.1)
Other	6 (8.3)	4 (5.6)

*Note*: Participants were randomly assigned to intervention (*n* = 72; psychoeducational programme) or control (*n* = 71; no psychoeducational programme) group. Unless otherwise specified, data are provided as number (percentage).

### Effects of ACT psychoeducation on MHS

**FIGURE 1:**
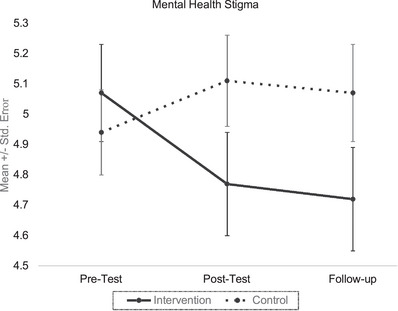
Levels of mental health stigma (MHS) in intervention versus control groups over time. *Note*: Effects of psychoeducational programme to reduce burden transfer on MHS in intervention and control groups at pre‐, post‐ and 1‐month follow‐up. The intervention group showed reduced MHS compared to baseline and the control group, with improvements maintained 1 month later. Error bars represent within‐subject 95% confidence intervals.

**FIGURE 2:**
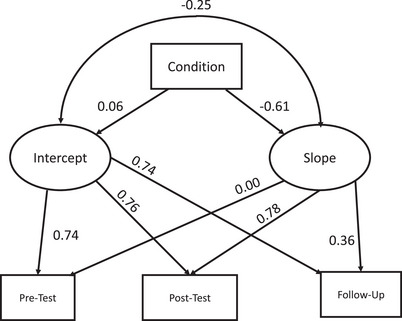
Longitudinal growth curve model. *Note*: Latent growth curve model with standardised parameter estimates. Fit indices suggest a good fit to the data *χ* ^2^(4) = 3.24, *p* = 0.52, *χ* ^2^/df = 0.81, root mean square error of approximation = 0.00 (90% confidence interval [0.00; 0.12]), standardised root mean squared residual = 0.03 and comparative fit index = 1.00.

See Figure 1 for descriptive statistics and Table 2 for percent agreement, neutral and disagreement with each MHS statement at each timepoint (participants with complete data only, *n* = 129). In the full sample (*n* = 143), a longitudinal growth curve model (see Figure 2) was specified with pre‐test, post‐test and follow‐up as the three time points to determine the intercept and slope, with condition (intervention vs. control) as a fixed covariate. All fit indices suggest that the model provided a good fit to the data, *χ* ^2^(4) = 3.24, *p* = 0.52, *χ* ^2^/df = 0.81, root mean square error of approximation = 0.00 (90% confidence interval [0.00; 0.12]), standardised root mean squared residual = 0.03 and comparative fit index = 1.00. In this model, condition predicted slope such that those who received the intervention reported less MHS over time relative to the control group, *β* = −0.28, *p* = 0.009. Condition did not predict the intercept, *β* = 0.12, *p* = 0.57, indicating that participants in the two conditions did not differ in initial MHS. Slope and intercept were not significantly associated (*r* = −0.25 *p* = 0.62) suggesting that initial levels of MHS were not associated with the rate of change over time.

**TABLE 2:** Percent responding agree/neutral/disagree with each mental health stigma statement (*n* = 129).

	Intervention group (*n* = 64)	Control group (*n* = 65)
Pre‐test	Agree/neutral/disagree	Agree/neutral/disagree
‘Treatment helps’	60 (93.8)/2 (3.1)/2 (3.1)	59 (90.8)/5 (7.7)/1 (1.5)
‘Caring towards’	16 (25.0)/6 (9.4)/42 (65.6)	20 (30.8)/12 (18.5)/33 (50.8)
Post‐test	Agree/neutral/disagree	Agree/neutral/disagree
‘Treatment helps’	57 (89.1)/7 (10.9)/0 (0)	60 (92.3)/5 (7.7)/0 (0)
‘Caring towards’	21 (32.8)/8 (12.5)/35 (54.7)	14 (21.5)/9 (13.8)/42 (64.6)
Follow‐up	Agree/neutral/disagree	Agree/neutral/disagree
‘Treatment helps’	57 (89.1)/6 (9.4)/1 (1.6)	60 (92.3)/4 (6.2)/1 (1.5)
‘Caring towards’	21 (32.8)/12 (18.8)/31 (48.4)	15 (23.1)/8 (12.3)/42 (64.6)

*Note*: Data are provided as number (percentage).

## DISCUSSION

The current study examined whether a psychoeducational programme designed to reduce stress and burnout related to difficult client interactions would also show a benefit of reducing MHS in veterinary healthcare teams. Relative to the baseline and a control group, those assigned to the intervention condition showed lower MHS immediately following the programme and 1 month later. The findings suggest that workplace training using evidence‐based psychoeducational methods that effectively improve mental health and wellbeing outcomes may also be an avenue for decreasing general MHS in this at‐risk group.

It is not fully clear whether exposure to evidence‐based psychological training specifically enhances veterinary healthcare providers’ opinions about mental health treatment, or if improvement in MHS is a product of a more generalised reduction in reactivity, as reduction in reactivity was an intended target of training. An important future direction will be examination of changes in mental health variables as potential moderating variables in the relationships presented in this work. Comparison of the programme described here versus a more typical ‘direct’ anti‐stigma programme^9^ would be important to examine in the future, although it is noted that the client interaction focus of the programme utilised here, with its obvious applicability to daily issues that are salient to veterinary teams, might have an advantage of being more palatable to veterinary teams relative to a programme attempting to directly operate on MHS. Additional follow‐up research should include longitudinal examination of the durability of these altered views on mental health and an investigation of whether the reductions in MHS noted here lead to a greater likelihood of seeking treatment for psychological problems.

Several strengths of the current work are highlighted, including the use of data collected utilizing a randomised controlled design, the inclusion of individuals across positions in veterinary medicine ranging from veterinarians to technicians and support staff, as well as recruitment across a variety of work environments from small animal general hospitals to emergency/referral clinics and a large academic centre. However, limitations are also acknowledged, most prominently, that individuals included in the current study self‐selected for participation in the psychoeducational training. This could bias results to reflect attitudes of individuals who were more psychologically open at baseline. However, relative to prior work in the general population (for which positive attitudes towards individuals with mental health problems and mental health treatment range from 37.0% to 93.0%),^15^ the current study demonstrated greater variability in positive endorsement of these items (21.5%–93.8%) with apparently lower belief in how caring people are towards individuals with mental illness. A prior study in veterinary medicine^3^ has utilised an adapted version of the MHS scale, making direct contrasts within the field more difficult to draw, but it does appear that the current sample was not particularly psychologically open. An additional limitation is that MHS was a secondary variable of interest in the current work. Larger studies with additional comparisons, explicit intention to study MHS and longitudinal designs of longer duration will be beneficial in determining the generalisability and durability of the current findings.

In conclusion, the current work examined the effectiveness of an evidence‐based ACT‐based psychoeducational programme, primarily intended to reduce burden transfer (namely reactivity to difficult client interactions) for reducing MHS in veterinary healthcare teams. As hypothesised, individuals assigned to the intervention group showed reduced MHS compared with both control and baseline, with improvements maintained 1 month later. The findings suggest that exposure to an evidence‐based psychological method that effectively improved mental health‐related outcomes may also reduce MHS in veterinary healthcare teams.

## AUTHOR CONTRIBUTIONS

Kylie R. Gannon conceived the presented idea and contributed to drafting of the manuscript. Elizabeth Cousins and Christopher Was contributed to statistical analysis and drafting of the manuscript. John Martin carried out the study. Alanna S.G. Updegraff and Mark D. Carlson contributed to intervention design. Mary Beth Spitznagel conceived the presented idea, supervised data collection and drafted portions of the manuscript. All the authors provided critical feedback and helped shape the research, analysis and manuscript.

## CONFLICTS OF INTEREST STATEMENT

The authors declare they have no conflicts of interest.

## FUNDING INFORMATION

The funding source was not involved in the study design, data analysis and interpretation, or writing and publication of the manuscript.

## ETHICS STATEMENT

Study protocols were approved by the Kent State University Institutional Review Board. The original research study was conducted in accordance with Consolidated Standards of Reporting Trials (CONSORT) guidelines.

## Data Availability

The data that support the findings of this study are available from the corresponding author (M.B.S.) upon reasonable request.
